# Comprehensive profiling of immune landscape changes and oxidative stress-associated biomarkers in intervertebral disc degeneration

**DOI:** 10.3389/fcell.2026.1979394

**Published:** 2026-10-29

**Authors:** Jianming Huang, Feng Gong, Jialin Li, Shihao Lu, Xiongfei Li, Jingxin Zhao, Yushu Bai, Xiaochuan Gu

**Affiliations:** 1 Department of Spine Surgery, Changhai Hospital, Naval Medical University, Shanghai, China; 2 Department of Anesthesiology, Changhai Hospital, Naval Medical University, Shanghai, China

**Keywords:** ceRNA network, immune infiltration, intervertebral disc degeneration, nucleus pulposus cells, oxidative stress

## Abstract

**Background:**

Intervertebral disc degeneration (IDD) is often considered a cause of low back pain. Oxidative stress and the dysregulation of the immune microenvironment are well recognized as important players in this pathology. However, the global knowledge about the core genes that are related to the oxidative stress and how they are involved in the immune response is still missing.

**Methods:**

We integrated transcriptomic data from three IDD cohorts (GSE167199, GSE176205, GSE186542) and performed differential expression analysis, WGCNA, consensus clustering, and CIBERSORT to identify oxidative stress-related genes, define molecular subtypes, and evaluate immune infiltration. We further constructed single-gene GSEA models, ceRNA networks, and TF-mRNA interactions. Biomarker validity was tested via qPCR and IHC in human samples, and immunofluorescence in a rat puncture model.

**Results:**

The study revealed two novel IDD subtypes with distinct oxidative stress signatures, and identified CD36, SESN3, and HMOX1 as key regulators. Their expression correlated with immune infiltration, particularly resting mast cells, naïve CD4^+^ T cells, and macrophages. GSEA linked them to cell cycle, metabolism, and cytokine pathways, while ceRNA and TF networks revealed complex regulation involving lncRNAs, miRNAs, and transcription factors. Validation confirmed their upregulation in human and rat degenerated discs, highlighting their diagnostic and therapeutic potential.

**Conclusion:**

The study used bioinformatics to identify and suggest CD36, SESN3 and HMOX1 as potential oxidative stress-associated biomarkers in IDD, and also underlines their strong relationship with the remodeling of the immune microenvironment. These results contribute to a better understanding of the relationship between oxidative processes and immune response in the context of the disc degeneration, and may help to conceive future therapeutic approaches with antioxidant and immunomodulatory effects.

## 1 Introduction

Intervertebral disc degeneration (IDD) is the main pathological basis for a series of degenerative spinal disorders. It is related to a chronic lower back pain, radiculopathy and spinal stenosis (Hammoor et al., 2026; Kirnaz et al., 2022). Because the global population is aging quickly, the clinical burdens and the economic cost related to IDD are increasing (van Os et al., 2025). Structurally, the intervertebral disc is a specific fibrocartilaginous entity with a sparsely vascularized network. In that tissue it is the nucleus pulposus cells (NPCs) which are responsible for the synthesis and the maintenance of the extracellular matrix (ECM) which is enriched in type II collagen and proteoglycans, in order to keep the disc hydration levels and mechanical stability (Chen et al., 2024; Cheng et al., 2025; Wang et al., 2025b). The homeostatic balance in the disc is gradually disturbed by several factors like the aging, an aberrant mechanical loading and a nutrient deprivation which leads to degenerative changes which are mostly irreversible (Silwal et al., 2023; Zhang et al., 2024). Among the different mechanisms of IDD, the oxidative stress has been considered as a major player for both the starting and the propagation of the pathology (Chang et al., 2025; Li et al., 2022; Zhu et al., 2023). Considering that the nucleus pulposus is located in a hypoxic niche coupled with high metabolic needs, it is also prone to the accumulation of reactive oxygen species (ROS) (Wang et al., 2025c; Zhang J. et al., 2025). While basal ROS concentrations are necessary for the proper intracellular communication process, their over generation perturbs redox homeostasis and induces the oxidative stress. High levels of ROS cause a direct damage to the lipids, proteins and DNA, but it also activates crucial signaling cascades like MAPK, NF-kB and PI3K/Akt pathways. All these events promote the senescence and apoptosis of the cells of both the annulus fibrosus and nucleus pulposus (Li B. et al., 2024; Li M. et al., 2024; Xie et al., 2024). At the same time, oxidative stress downregulates the anabolic activities and upregulates the synthesis of matrix degrading enzymes like ADAMTS family members and matrix metalloproteinases (MMPs). This shift will accelerate the breakdown of the extracellular matrix (ECM) components (Chen et al., 2019; Hua et al., 2025; Liu et al., 2024; Zhao et al., 2011).

In particular, the main ECM components are targeted as, for example, aggrecan and collagen type II,Moreover, this process also enhances the chronic inflammatory response by activating the NLRP3 inflammasome pathway, which then drives the secretion of pro-inflammatory mediators, such as tumor necrosis factor-alpha (TNF-α), interleukin-6 (IL-6), and interleukin-1 beta (IL-1β) [23–25]. These events push the disc microenvironment in a vicious cycle characterized by oxidative stress, persistent inflammation and progressive tissue degradation. Thus, IDD is not anymore interpreted as a consequence of a cellular malfunction inside the disc. In fact, an increasing attention is given to the alteration of the local immune milieu during the degenerative progression. Under physiological healthy conditions, the intervertebral disc is considered as an immune-privileged site. Nevertheless, when the degeneration progresses, a compromise of the endplate barrier, with pathological vascularization and nerve growth, promote the invasion of immune cells, in particular macrophages and T lymphocytes, in the disc matrix (Jin et al., 2026; Li N. et al., 2026; Mao et al., 2026; Song et al., 2023; Wu et al., 2024). Upon the entry of these immune cells.

These cells will interact with the native disc cells upon their infiltration, and thus, will promote the degenerative cascade. It is important to underline that also the oxidative stress plays a key role in the modulation of the recruitment, the functional activity and the polarization of the immune cells, in particular of the macrophages (Xu et al., 2023; Zhang C. et al., 2025). In fact, the reactive oxygen species (ROS) would push the macrophages to become the pro-inflammatory M1 phenotype. A change in phenotype leads to an increased production of the both of ROS and inflammatory cytokines, that will aggravate the tissue injury (Chen et al., 2023; Risbud and Shapiro, 2014; Yang et al., 2020; Zhou et al., 2024). Thus, a detailed analysis of the molecules associated to the oxidative stress within the immune regulatory framework of the IDD is necessary to have a better insight on the pathogenesis of the condition.

A lot of literature in the increasing body have pointed out the participation of oxidative stress associated genes in the intervertebral disc degeneration (Chen et al., 2026; Pumputis et al., 2025; Wang et al., 2025a). However, for the previous investigations, the datasets or the cohort were studied in an isolated manner with not a too much sample size. This fact has limited the elucidation of the important molecular networks that can connect oxidative stress to the immune regulation in IDD, which limits the progress of the identification of robust biomarkers and the design of targeted therapeutic strategy. In this problem, in our research we had gathered several transcriptomic dataset in the GEO database related to IDD and we performed a comprehensive bioinformatics analysis. We combined the differential expression analysis with the weighted gene co-expression network analysis (WGCNA) and we found the oxidative stress related genes that have in a matching way a change in the degenerative disc. We also had examined the correlation with these genes and the patterns of the immune cell infiltration, and we also built the network on the base of the ceRNA interactions and with the transcription factors.

We constructed the regulatory networks in order to understand the upstream mechanisms of these processes. In the last step, we confirmed the expression level of these hub genes by human disc tissue sample and rat disc degeneration model. All these investigations will increase our understanding about the molecular interplay between the oxidative stress and the immune remodeling in IDD, which can help in the finding of the valid diagnostic biomarkers and therapeutics.

## 2 Materials and methods

### 2.1 Data acquisition and preprocessing

Three transcriptomic datasets related with intervertebral disc degeneration, namely GSE167199, GSE176205 and GSE186542, were downloaded from the Gene Expression Omnibus (GEO) repository. All these collections were composed by 21 samples, 9 from the healthy nucleus pulposus tissue and 12 from the degenerative nucleus pulposus condition. The datasets were put together at first to have a better set of data and be more reliable of the following analyses. Then, the ComBat algorithm was used to correct for the batch effect due to the technical variations between the different sources. This was necessary to reduce the discrepances and make the data more comparable to make a more strict examination of the transcriptomic profiles related with the intervertebral disc degeneration.

### 2.2 Identification of differentially expressed genes and oxidative stress-related candidate genes

In order to study the variations in the expression of genes for the *a priori* defined cohorts, we used the limma package in the R environment, which is known for its use in bioinformatics in the treatment of transcriptomic data. In the present investigation, we considered differentially expressed genes (DEGs) by following very strict statistics thresholds which are an adjusted p-value smaller than 0.05 and an absolute log2 fold change larger than 0.5. Such strict criteria will ensure that the following analyses will be done with only genes that have a statistically significant alteration of the levels of expression. To extract the genes specifically associated with the oxidative stress, the resulting DEGs were intersected with a particular collection of oxidative stress associated genes taken from the Molecular Signatures Database (MSigDB). Such an overlapping will allow to get the genes that are marked differentially expressed but also that have a biological meaning with oxidative stress mechanisms. The genes found in the last part of the analysis were named candidate genes associated with oxidative stress, which can be used to carry out more complete studies of its functions and importance in the related biological mechanisms.

### 2.3 Consensus clustering analysis

In order to elucidate the molecular diversity related to oxidative stress in the context of the intervertebral disc degeneration (IDD), we used a consensus clustering approach on the basis of the expression pattern of oxidative stress related genes. The determination of the optimal number of clusters needed a strict check, including the study of the delta area plots, the cumulative distribution function (CDF) and the stability of the consensus matrix. In order to be sure that we have a good investigation, we limited the maximum number of cluster to eight. The groups obtained were then plotted and checked using t-distributed stochastic neighbor embedding (t-SNE), which allow a particular depiction of these clusters in a less dimensional space. After the clustering phase, the differential expression analysis was done to compare the gene expression profile on the groups found, using the limma package for this.

The microarray data was analyzed by the well established instrument for linear modeling, the limma package. In order to understand the biological meaning of the differentially expressed genes, the Gene Ontology (GO) enrichment analysis was done by using the package called clusterProfiler. This procedure allows to better understand the functional roles and the pathways related to the genes in the different clusters related to oxidative stress in the intervertebral disc degeneration (IDD).

### 2.4 Weighted gene Co-expression network analysis

The Weighted Gene Co-expression Network Analysis (WGCNA) approach was applied to find the gene modules related to the oxidative stress related subtypes. Firstly, we removed the possible outlier samples to make sure the precision of following analytical procedures. Then a suitable soft-thresholding power was chosen by following the rules of scale-free topology, which is very important to build a stable and reliable network architecture. Then an adjacency matrix and a topological overlap matrix were generated, which is the basic for investigating the inter-genic relationship. The genes were well categorized into separate co-expression modules by the application of hierarchical clustering techniques. To find out the relations of these modules with clinical features, the correlations were calculated between module eigengenes and different clinical traits. The main goal of this evaluation is to find the key modules which show the significant relevance to the clusters related with the oxidative stress. In this analytical context, the genes showing high module.

Intramodular hub genes were found by checking for both module membership and gene significance. We expect these genes to have a major impact on the biological mechanisms related to oxidative stress and they will be important hubs for further studies in this area.

### 2.5 Single-gene gene set enrichment analysis

For all the identified hub gene, the samples were divided into high-expression and low-expression cohort for each gene according to the median of expression. The rank list of genes was then made according to the differential expression pattern of the two groups. We checked the pathways related to each single hub gene by doing gene set enrichment analysis (GSEA) by using the gseKEGG function in the clusterProfiler package. The most enriched pathways were plotted by using the gseaplot2 function.

### 2.6 Construction of ceRNA and TF-mRNA regulatory networks

#### 2.6.1 miRNA-mRNA interaction analysis

By using the multiMiR R package, that collects the information of different authoritative repositories, among which are miRTarBase and TarBase, the binding events between miRNAs and mRNAs associated with hub genes, that were experimentally validated, have been retrieved.

#### 2.6.2 lncRNA-miRNA interaction analysis

In order to build the regulation with the lncRNAs, we considered the human lncRNA-miRNA pairs with experimental evidence from the NPInter database (version 4.0).

#### 2.6.3 ceRNA network construction

By the combination of the verified binding pairs of mRNA-miRNA and of miRNA-lncRNA, we built a holistic three-layer competitive endogenous RNA (ceRNA) regulatory framework. Furthermore, we did the streamlining procedure in order to make the network more interpretable and biologically more relevant. The refinement strategy we applied will keep only the fifteen more important miRNAs related to every mRNA, and the five lncRNAs with most validation for each related miRNA.

#### 2.6.4 TF-mRNA regulatory network construction

The experimentally confirmed interactions between the transcription factors (TFs) and the targets were obtained using the dorothea R package and stratified into three levels of confidence: A, B and C. Using this dataset, a directed regulatory network was built to define the relations between the TFs and the mRNA to find the possible upstream transcriptional regulators that are related to the hub genes of the study. In order to plot the competing endogenous RNA (ceRNA) network, a concentric circular arrangement was done by using the ggraph package. On the other hand, the Fruchterman-Reingold layout algorithm was used to plot the TF-mRNA regulatory network, which provides a clear picture of the complicated interactions of this system.

### 2.7 Immune infiltration analysis

The CIBERSORT deconvolution technique was performed with the deconvo_tme function included in the IOBR R package (version 0.99.9) in order to get the proportion of the twenty two different immune cell populations in each of the analyzed specimens. The analytical framework was set with the following arguments: the algorithm is set to “cibersort”, the array is included (as TRUE) and the number of permutations is set to 100. In order to not have any cell fraction taken as negative, those calculated which were negative were set to 0. Stacked bar charts were made to show the immune landscape in the different molecular clusters and disease classification in order to show which are the different patterns of immune infiltration of the groups studied. Also the Wilcoxon rank-sum test was used to make a strict statistical test to these variations, showing which is how immune cell abundance is different according to the group that we put in. In addition, we also used Spearman correlation analysis to explore the relationships among different immune cell populations, and the relationship between the expression levels of hub genes and the abundance of immune cells. The significant correlations were visualized by building heatmaps using the corrplot package, and only the significant results were shown.

### 2.8 Validation of candidate biomarkers in human tissues

In order to test the candidate biomarkers at the messenger RNA (mRNA) stage, the quantitative polymerase chain reaction (qPCR) assays were performed on the human disc tissue specimen. These samples were from subjects that were diagnosed with the intervertebral disc degeneration (IDD), as well as from the control subjects without this condition. Every participant has given written informed consent in accordance with the ethical guidelines before being included in the study. The tissue sample was collected only on the patients who were in the spinal fusion procedure, which guarantee the obtaining of the relevant and true biological material. The cohort of the study was 9 participants, which included 5 men and 4 women. The preoperative magnetic resonance imaging (MRI) was used to check the severity grade of lumbar disc degeneration.

Participants classified as Grade II were assigned to the control cohort and participants presenting with Grades III, IV and V constituted the IDD group. For each grade category three tissue specimens were obtained. Total RNA isolation was done using TRIzol reagent provided by Invitrogen, according to the instructions of the manufacturer. We measured RNA concentration precisely by a Bio-Tek microplate reader to assure quantification. Reverse transcription was carried out using oligo-dT primers obtained from Takara (Kusatsu, Japan). Quantitative PCR amplification was done using SYBR Green Premix Ex Taq polymerase, also obtained from Takara. Data were normalized against GAPDH and relative mRNA expression levels were determined using the 2^−ΔΔCt^ computational approach to analyse data. The primer sequences used were as follows: CD36(F:5′-AAGCCAGGTATTGCAGTTCTTT-3′, R: 5′-GCA​TTT​GCT​GAT​GTC​TAG​CA-3′); SESN3(F:5′-TACATCCGGTCGTCTGG AC AA-3′,R:5′-AAAACTGGCTCCGCAAGAAAG-3′); HMOX1 (F: 5′-AAGACT GCG TTCCTGCTCAA-3′, R: 5′-AAAGCCCTACAGCAACTGTCG-3′),GAPDH (F: 5′-GGA​GCG​AGA​TCC​CTC​CAA​AAT-3′, R: 5′-GGC​TGT​TGT​CAT​ACT​TCT​CAT​GG-3′). Nucleus pulposus specimens were fixed with 4% paraformaldehyde to perform immunohistochemical examination. The tissue samples were then dehydrated, embedded in paraffin wax, and sliced with the thickness of about 5 μm. Sections were treated with primary antibodies at 1:200 dilution with CD36 (Catalog num:66395-1-Ig, Proteintech, China), SESN3 (Catalog num:11431-2-AP, Proteintech, China), and HMOX1 (Catalog num:66743-1-Ig, Proteintech, China). The section was incubated overnight. After the primary antibody, the sections were treated with secondary antibody diluted at 1:500 for 2 hours at room temperature. An enhanced DAB kit was used to show immunoreactive signals to detect the protein of interest. ImageJ software was used for the quantitative staining intensity test.

### 2.9 Rat model of IDD and immunofluorescence validation

The study protocol was ethically cleared by the Animal Ethics Committee at Changhai Hospital, Naval Medical University. All the experimental steps were following the ARRIVE guidelines. Male Sprague-Dawley rats (12 weeks old, 300–350 g) were kept in specific pathogen free environment during the 1 week acclimatization period before the surgery. Anesthesia was given by intraperitoneal injection of 1% sodium pentobarbital. The tail region was thoroughly disinfecterd and the target intervertebral disc was identified by palpation. A sterile 26-gauge needle was put vertically into the center of the disc, advanced about 5 mm into nucleus pulposus, rotated 360°, and maintained in the position for 30 s to create a standard mechanical injury. For the sham-operated control group, the tail was exposed in the same way; According to the protocol reported in previous studies, caudal vertebral segments were taken after a 4 week postoperative interval of recovery (Poletto et al., 2022). The samples were stored in a 4% paraformaldehyde fixative and decalcified by immersion in 10% EDTA. The tissues were embedded in paraffin wax and cut in sections with 4–5 μm of thickness. The slides were deparaffinized and rehydrated and then the antigen retrieval was done using preheated EDTA buffer. The sections were permeabilized with 0.5% Triton X-100 and blocked with the mixture of goat serum and bovine serum albumin (BSA). The primary antibodies were used diluted 1:200 and incubated one night. This step was followed by the addition of the fluorescently labeled secondary antibodies (diluted 1:500) that were let react 1 hour at ambient temperature in dark. Nuclear staining was done with DAPI, and Laser scanning confocal microscopy was employed to acquire images for subsequent examination. Evaluate the Histology Grade according to the previously reported method (Lai et al., 2021).

### 2.10 Statistical analysis

The tasks of data management, statistical evaluation and figure creation, were done with SPSS (version 23), GraphPad Prism 6 and R (version 4.3.2). For the case of continuous variables distributed according to a normal distribution, comparisons were done with independent-samples t-test and the statistical significance was set at p below 0.05.

## 3 Results

### 3.1 Identification of oxidative stress-related differentially expressed genes in IDD

The transcriptomic data were aggregated from 3 different GEO datasets, with 9 control and 12 IDD cases in total. Before the batch effect correction, the samples from different dataset obviously clustered separated, which suggests the existence of considerable technical variable (Figure 1A). However, the ComBat algorithm could well reduce this systematic bias (Figure 1B). The principal component analysis on the corrected data already showed a clear divergence between the control and IDD group, which supported the existence of disease specific transcriptional alterations (Figure 1C). After the normalization procedures, the expression pattern of all samples was uniform (Figure 1D). Also, the differential expression profiling found a large cohort of genes were either significantly up or down when compared the two groups (Figure 1E). Moreover, the functional enrichment assessments showed that these differentially expressed genes (DEGs) have strong correlations with the biological processes related to oxidative stress as explored by Gene Ontology (GO) analysis (Figure 1F). On the other hand, KEGG pathway analysis showed the significant enrichment in several signaling cascades, such as the MAPK pathway (Figure 1G).

**FIGURE 1:**
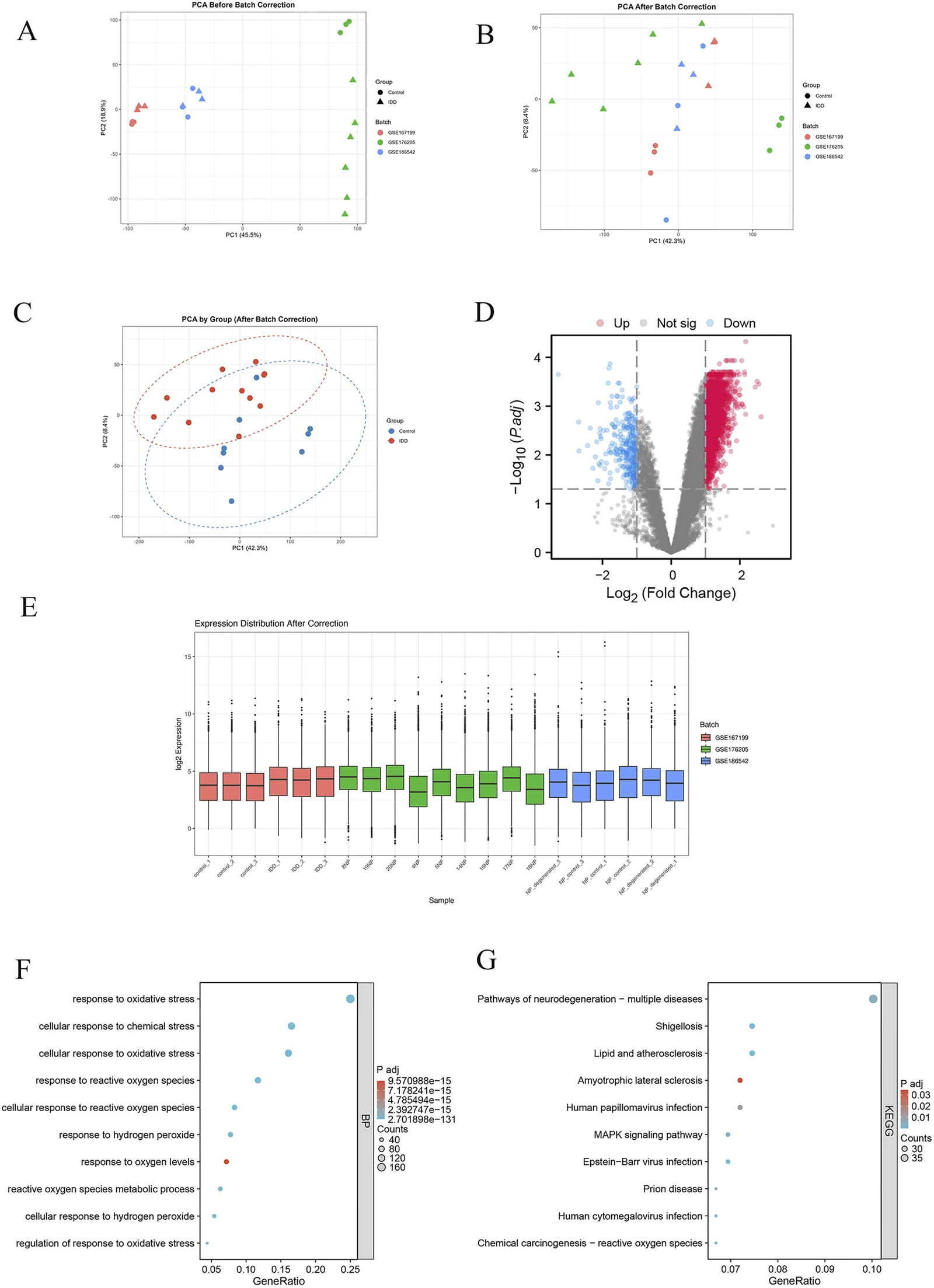
Identification of oxidative stress-related differentially expressed genes in intervertebral disc degeneration. **(A)** Principal component analysis (PCA) plot before batch correction, showing sample separation according to dataset source. **(B)** PCA plot after ComBat-based batch correction. **(C)** PCA plot grouped by disease status (control versus IDD), showing a separation trend between groups. **(D)** Boxplot of normalized gene expression values across all samples. **(E)** Volcano plot of the differentially expressed genes when comparing the control group with the IDD cohort. **(F)** Gene Ontology enrichment analysis of differentially expressed genes. **(G)** KEGG pathway enrichment analysis of differentially expressed genes.

### 3.2 Consensus clustering revealed distinct oxidative stress-associated molecular subtypes

In order to explore the heterogeneity related to oxidative stress in the setting of IDD, we perform a consensus clustering on the cohort of 21 samples. The consensus matrix, as well as the cumulative distribution function (CDF) curves and the delta area plots, all pointed out that k = 2 between Cluster 1 and Cluster 2. Under such a parameter, the consensus heatmap has a clear diagonal block structure, which means that we have a strong partitioning of the samples into two different molecular subtypes, as shown in Figures 2A–C. The validity of such a categorization was also supported by t SNE analysis, where we can see a clear spatial separation between the two considered clusters (Figure 2D). Then by doing the differential expression analysis between Cluster 1 and Cluster 2, we found a set of genes with important expression different. By referring to a list of oxidative stress genes given in advance, we were able to get some oxidative stress related differentially expressed genes (OSRDEGs), as shown in Figures 2E,F. Finally, the Gene Ontology (GO) enrichment analysis showed that these genes are mainly related with redox homeostasis, metabolism of fatty acids, and cellular responses to oxidative stress, which are closely related with the pathophysiology of disc degeneration (Figure 2H). It is interesting to note that CD36, SESN3 and HMOX1 were among the center hub genes of the top OSRDEGs, with clear difference on expression levels of the two subtypes (Figure 2G).

**FIGURE 2:**
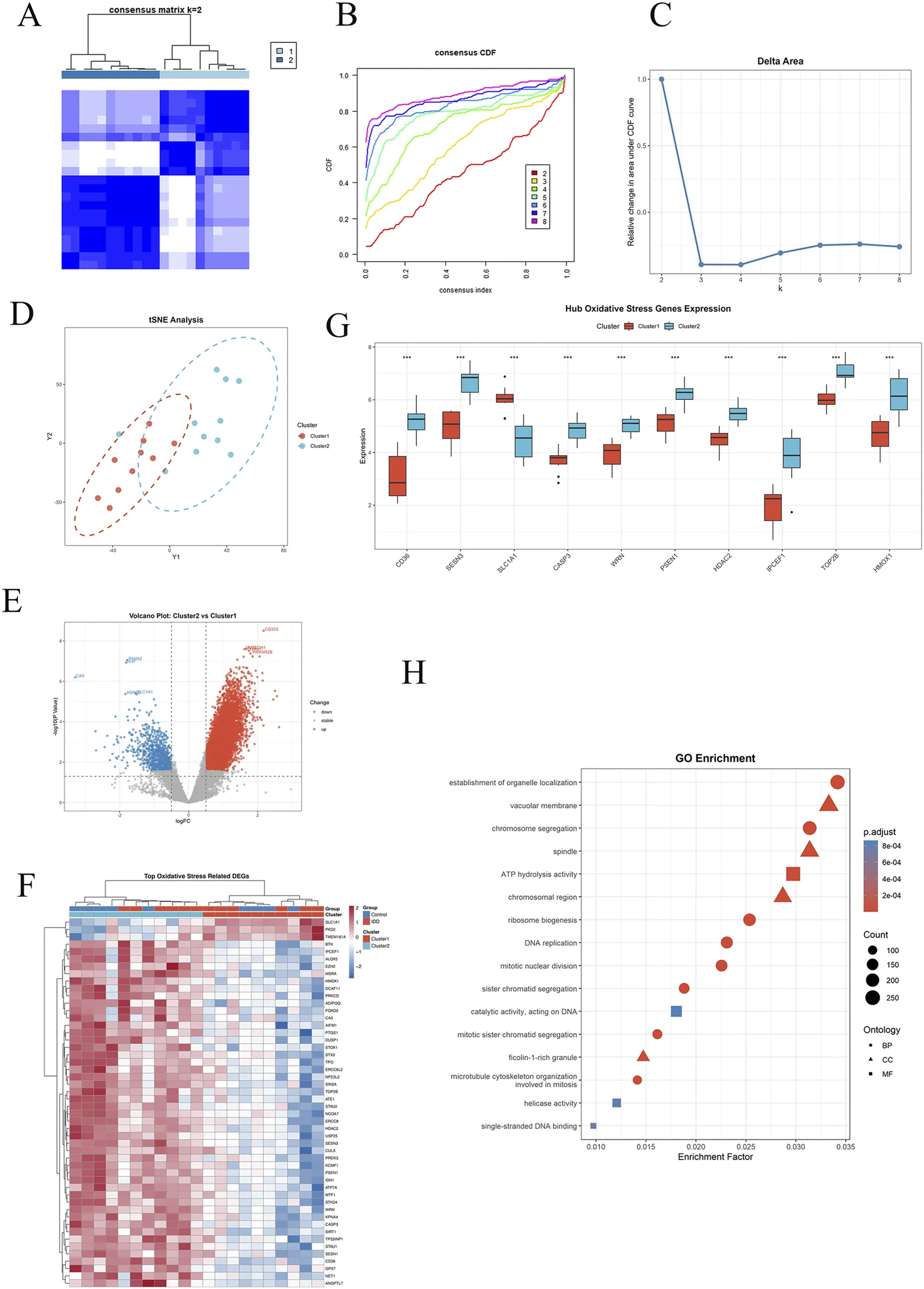
Consensus clustering identifies oxidative stress-related molecular subtypes in IDD. **(A)** Consensus matrix heatmap for k = 2, showing stable clustering of samples into two subtypes. **(B)** Cumulative distribution function (CDF) curve used to evaluate clustering stability across different values of k. **(C)** Delta area plot supporting k = 2 as the optimal number of clusters. **(D)** t-SNE plot demonstrating clear separation between Cluster 1 and Cluster 2. **(E)** Volcano plot of differentially expressed genes between the two clusters. **(F)** Bar plot of the top 10 oxidative stress-related differentially expressed genes. **(G)** Expression boxplots of representative hub genes. **(H)** GO enrichment analysis of cluster-associated differentially expressed genes.

### 3.3 WGCNA identified a key module associated with oxidative stress subtypes

In order to identify genes related to oxidative stress-linked subtypes, we used Weighted Gene Co-expression Network Analysis (WGCNA). We did not see any prominent outliers in the initial hierarchical clustering of the samples. We could therefore add the clinical variables, like the disease state and subtype categorization, to the resulting dendrogram (see Figure 3A). We chose the appropriate soft-thresholding power by looking at both the scale-free topology fit index and mean connectivity (Figure 3B). With this good threshold we built the co-expression network and we assign the genes to diferent modules, that are characterized by a different color (Figure 3C). In the view of the associations between module eigengenes and clinical traits, the module in turquoise (2 539 genes) had the strongest association with the oxidative stress related subtype phenotype (Figure 3D).

**FIGURE 3:**
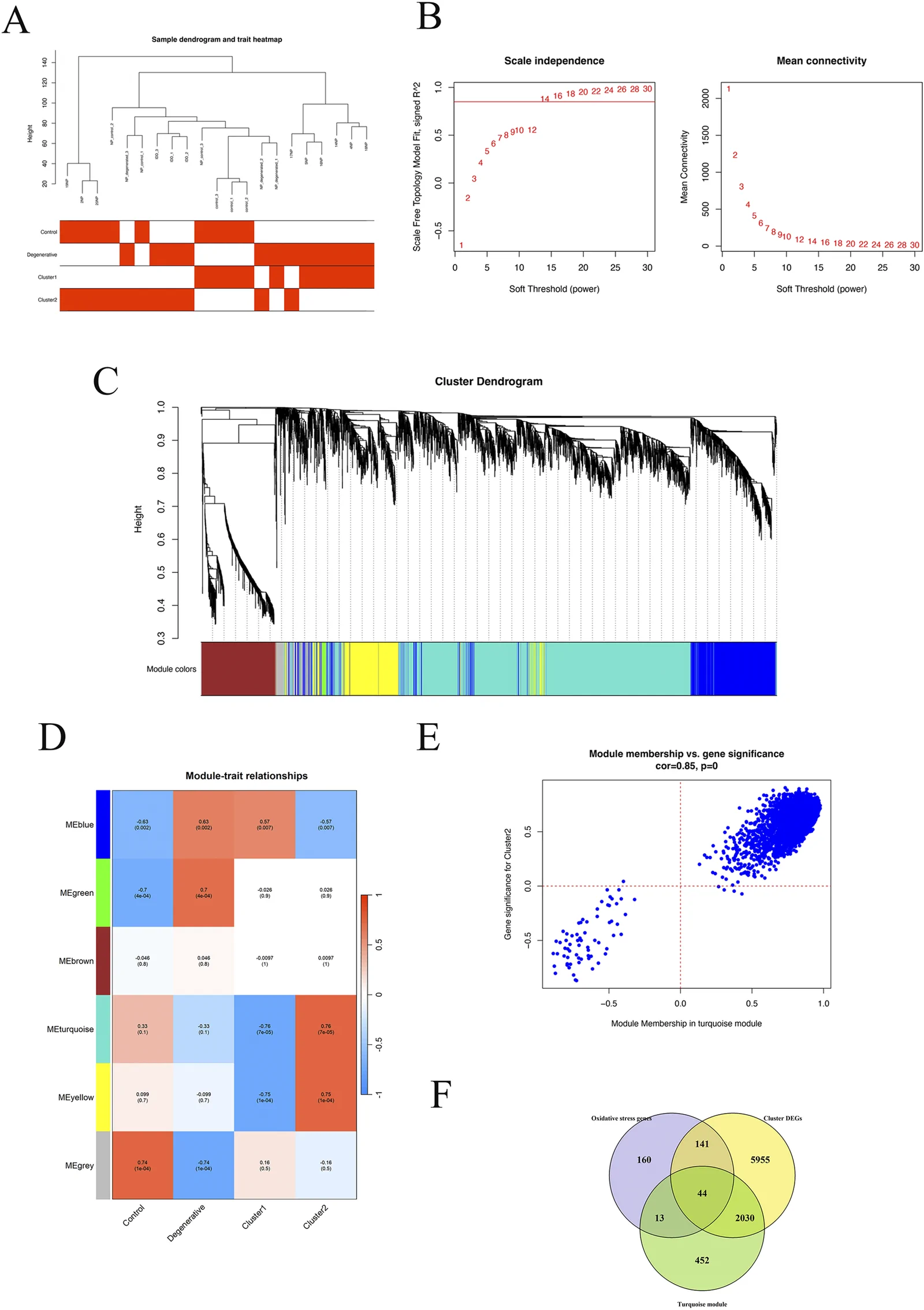
WGCNA identified a gene module associated with oxidative stress-related subtypes. **(A)** Sample clustering dendrogram with corresponding trait heatmap. **(B)** Analysis of scale independence and mean connectivity for soft-threshold selection. **(C)** Gene dendrogram showing module identification by dynamic tree cutting. **(D)** Heatmap of correlations between module eigengenes and clinical traits. **(E)** Scatter plot showing the relationship between gene significance and module membership in the key module. **(F)** Venn diagram of genes from the key module, genes of the differentially expressed genes belonging to the clusters and genes of oxidative stress.

The correlation that we observed between gene significance and module membership indicates the biological importance of these genes (Figure 3E). If we set the gene significance criterion as above 0.6, and the module membership as over 0.7, we finally selected in total 44 hub genes in the modules (Figure 3F).

### 3.4 Screening of hub biomarkers and construction of regulatory networks

By merging the 44 hub genes in the modules that are obtained by WGCNA with the main DEGs related to the oxidative stress obtained by the cluster, three common candidate biomarkers are found: CD36, SESN3 and HMOX1 (Figure 4A). To investigate the biological meaning of these particular genes we did single gene GSEA. The result was that expression of SESN3 was negatively related to the pathway of interaction between viral proteins and cytokine receptors. CD36 was negatively related to ascorbate and aldarate metabolism, the cell cycle, the Fc epsilon RI signaling, the ubiquinone and other terpenoid-quinone biosynthesis, the interaction between viral proteins and cytokines and the interaction between viral proteins and receptors. HMOX1 was negatively related to the cell cycle, the fructose and mannose metabolism and the interaction with neuroactive ligands and interactions with receptors, the progesterone-driven maturation process of oocytes, as well as the biosynthetic pathways for ubiquinone and other terpenoid-quinones (Figures 4B– 4D). To explore the possible upstream regulation mechanisms, we built a ceRNA network by including the experimentally validated interactions between mRNA-miRNA and miRNA-lncRNA pairs. The network analysis indicates that the three hub genes could be regulated in a complex post transcriptional way by different kinds of noncoding RNAs (Figure 4E). We also built a TF-mRNA regulatory network to find the transcription factors that may potentially regulate the expression levels of CD36, SESN3 and HMOX1 (Figure 4F).

**FIGURE 4:**
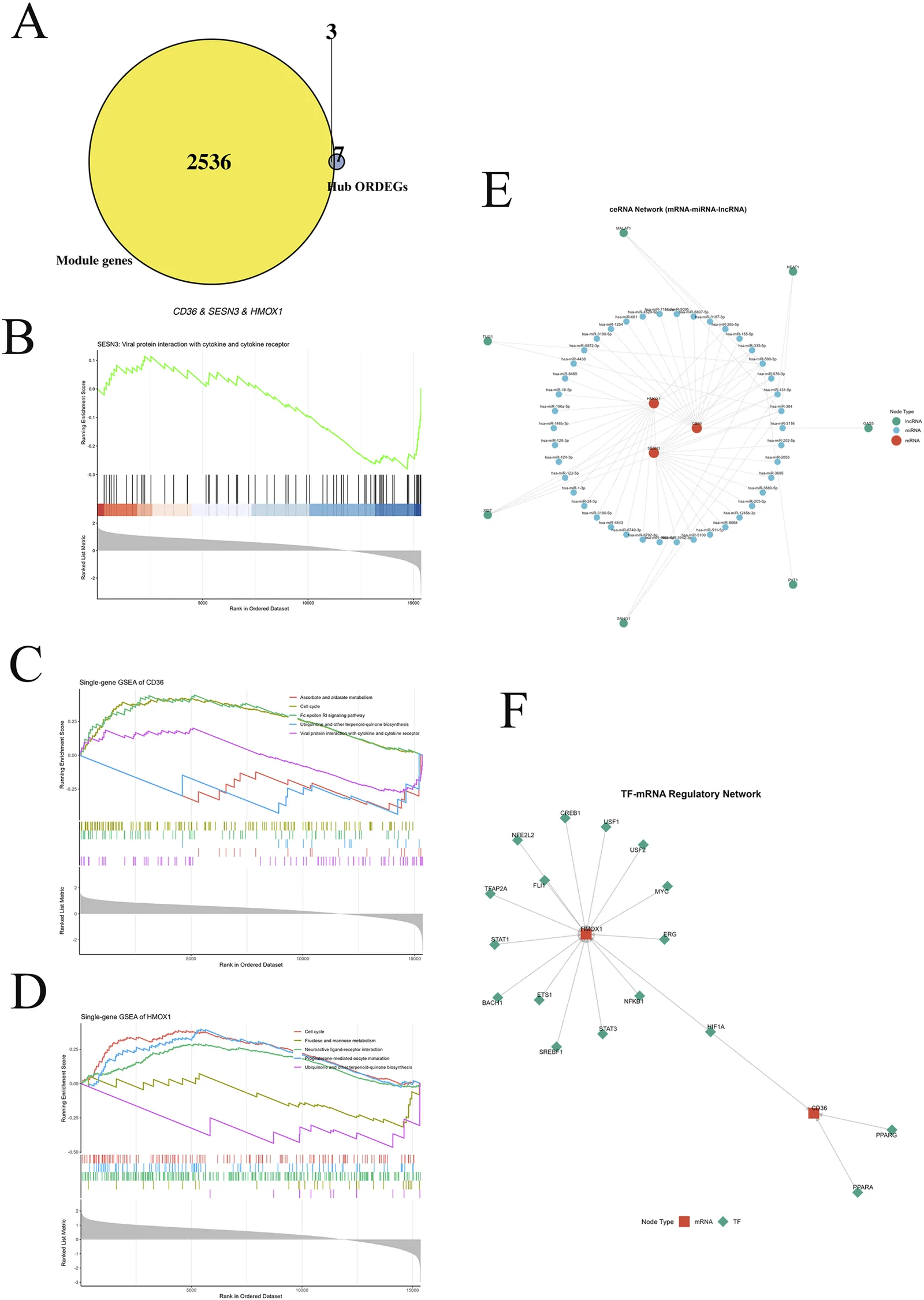
Functional enrichment and regulatory network analyses of hub biomarkers. **(A)** Venn diagram of the overlap of the hub genes which are found by WGCNA and the differentially expressed genes related to oxidative stress. **(B–D)** Single-gene GSEA plots for SESN3 **(B)**, CD36 **(C)**, and HMOX1 **(D)**, respectively. **(E)** ceRNA regulatory network of the three hub genes. **(F)** TF-mRNA regulatory network of the three hub genes.

### 3.5 Immune infiltration characteristics and correlation with hub genes

The CIBERSORT-based assessment, showed the specific immune infiltration profile related to different molecular subtypes. For example, we can see that the abundance of resting mast cells is much higher in cluster 2 comparing to cluster 1, but the prevalence of naive CD4^+^ T cells is much lower in this cluster (Figures 5A–C). For the disease status, the IDD cohort has the decrease proportion of resting memory CD4^+^ T cells compared with control (Figures 5B–D). Furthermore, Spearman correlation analysis showed the complex interplay among different infiltrating immune cell types (Figure 5E). Activated NK cells were negatively correlated with naive CD4^+^ T cells, plasma cells and resting NK cells. M1 macrophages was inverse with resting NK cells but positively related with activated NK cells. Activating dendritic cells was positive related with CD8^+^ T cells and activated memory CD4^+^ T cells. We performed an analysis between hub gene expression level of and the infiltration of immune cells, we found that the expression of HMOX1 is positively associated with the resting mast cell, the M0 macrophage and the activated NK cell, while it is negatively associated with the naive CD4^+^ T cell and the naive B cell. For CD36, we only see a positive association with the resting mast cells. Similarly, for SESN3 we see a positive link to the resting mast cell, but an inverse link to the naive CD4^+^ T cell, see Figure 5F. These results indicate that the biomarkers of oxidative stress are related to the changes of the immune micro environment in the IDD.

**FIGURE 5:**
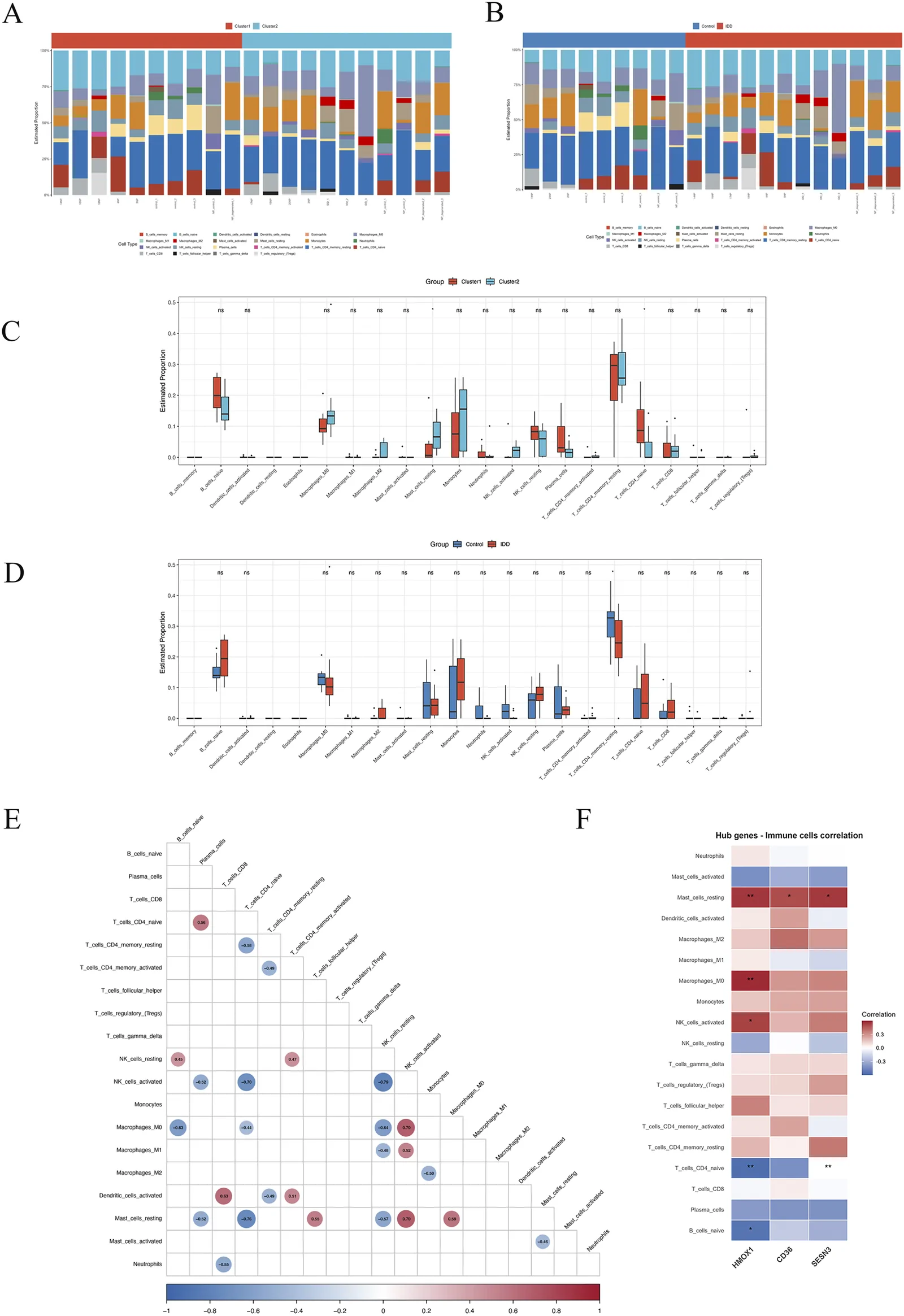
Immune infiltration landscape and its association with hub genes in IDD. **(A)** Stacked bar chart showing the relative abundance of 22 immune cell populations in each sample, split by molecular subtype (Cluster 1 vs. Cluster 2). **(B)** Stacked bar plot showing immune cell composition grouped by disease status (Control vs. IDD). **(C)** Boxplots comparing immune cell proportions between Cluster 1 and Cluster 2. **(D)** Boxplots comparing immune cell proportions between Control and IDD groups. **(E)** Spearman correlation heatmap among 22 infiltrating immune cell types. **(F)** Correlation heatmap showing the relationships between hub gene expression (HMOX1, CD36, SESN3) and immune cell abundance.

### 3.6 Experimental validation of hub genes in human tissues

In order to validate the biomarkers identified in this work, a cohort of six nucleus pulposus tissue specimens was collected, containing three control samples from people with Pfirrmann grade II discs and three degenerative samples from patients with Pfirrmann Grade III, IV, and V discs (Figures 6A,B). The immunohistochemical staining results show that CD36, SESN3 and HMOX1 are upregulated in the degenerated discs compared with controls (Figures 6C–F). Also, the quantitative polymerase chain reaction (qPCR) assessment also substantiates these observations, showing that the mRNA expression level for all three genes are much higher for the degeneration group compared with the control group (Figures 6G–I). Nucleus pulposus cells isolated from tissues at different degenerative stages were divided into control and IDD groups according to previous grouping. The results showed increased expression of SESN3, CD36, and HMOX1 in cells derived from degenerated nucleus pulposus (Figures 6J–M).

**FIGURE 6:**
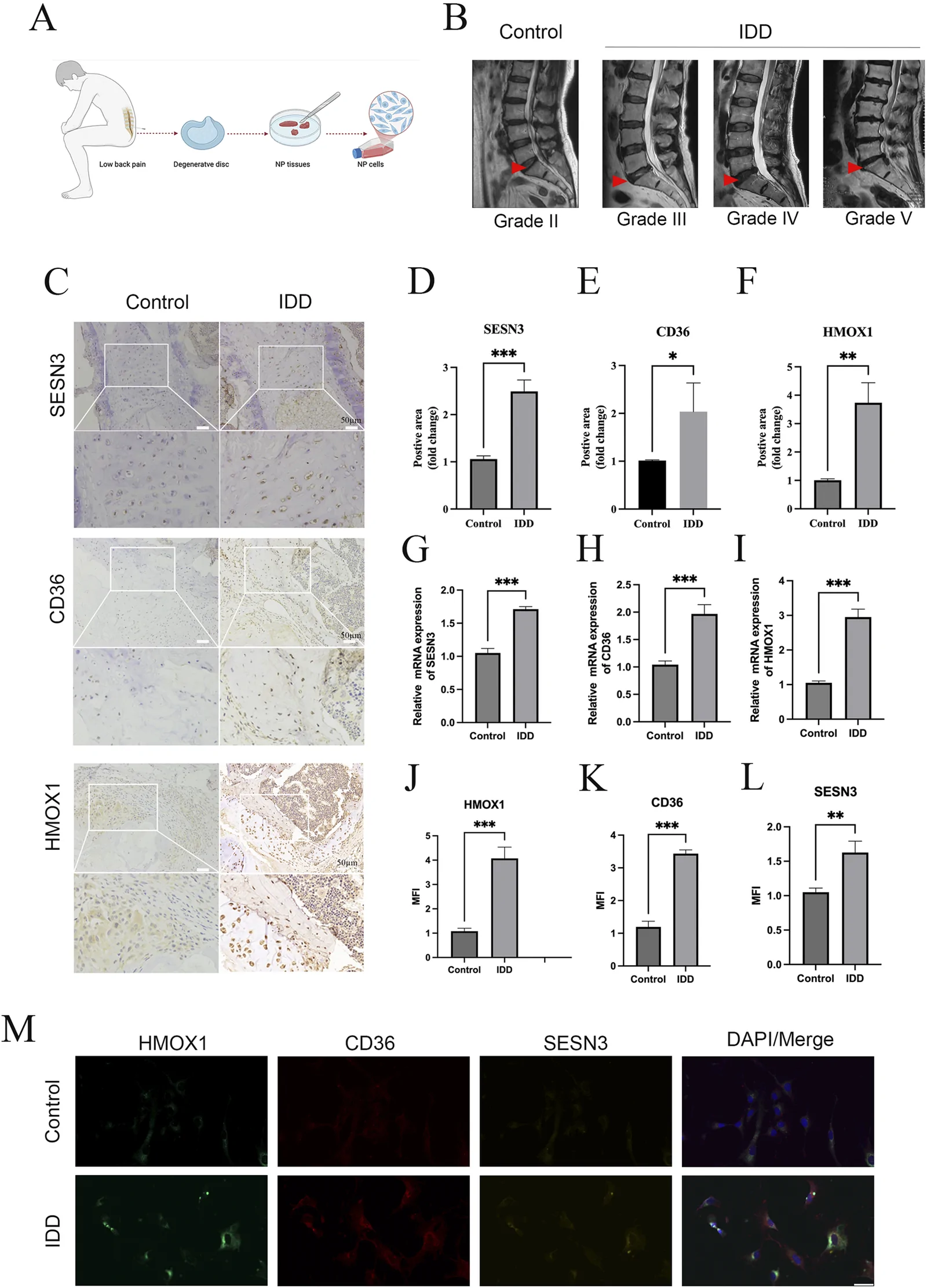
Validation of hub genes in human disc tissues. **(A)** Schematic illustration of tissue and cell acquisition. **(B)** Representative MRI images of intervertebral discs at different degenerative stages. **(C)** Representative immunohistochemical staining of CD36,SESN3 and HMOX1. **(D)** Quantitative analysis of SESN3 immunohistochemical staining. **(E)** Quantitative analysis of CD36 immunohistochemical staining. **(F)** Quantitative analysis of HMOX1 immunohistochemical staining. **(G–I)** qPCR detection of the expression levels of CD36, SESN3, and HMOX1 in the control and IDD specimens. **(J–L)** Quantitative immunofluorescence analysis of CD36, SESN3, and HMOX1 in nucleus pulposus cells between the control group and IDD group (Grade III -V). **(M)** Representative image of immunofluorescence staining. ∗P < 0.05, ∗∗P < 0.01, ∗∗∗P < 0.001.

### 3.7 Increased expression of CD36, SESN3, and HMOX1 in a rat model of intervertebral disc degeneration

Finally, in order to further validate these results in an *in vivo* situation, a rat model of intervertebral disc degeneration (IDD) was performed via needle. We used the puncture method to build the model. The histological examination of the tissues of the experimental group, such as H&E, Masson and SOFG staining, showed obvious degenerative changes in the intervertebral discs of the experimental group, especially the obvious loss of the nucleus pulposus tissue (Figures 7A,D). The immunofluorescence assay also showed that the expression levels of CD36, SESN3 and HMOX1 are much upregulated in the degenerated discs of the rat than that in the sham-operated group (Figures 7B,C), these results also support the reliability of the bioinformatics screening strategy used in this research. qPCR showed that the mRNA expression level for all three genes are much higher for the degeneration group compared with the control group in Rat models (Figures 7E–G).

**FIGURE 7:**
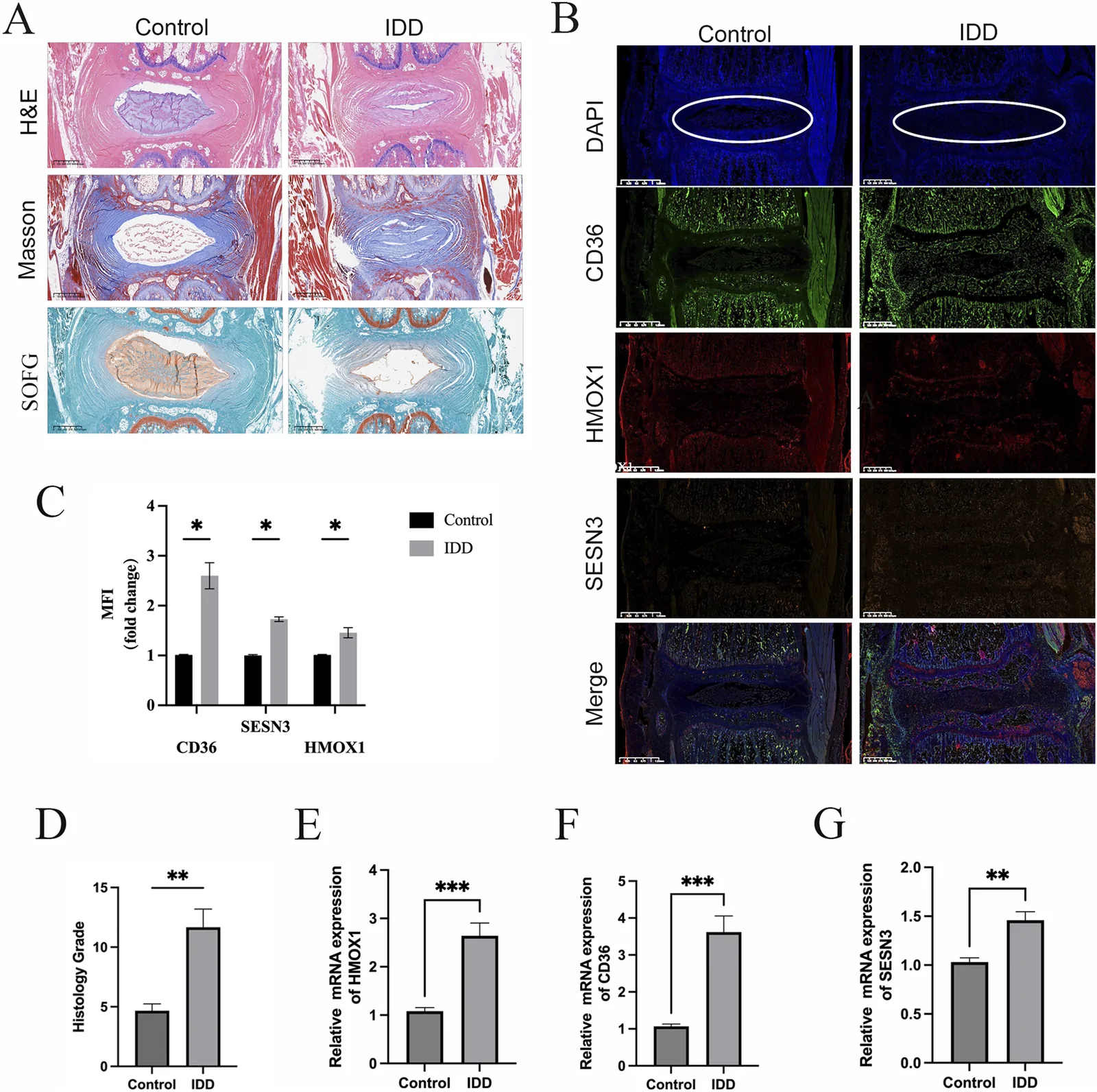
Increased expression of CD36, SESN3, and HMOX1 in a rat model of intervertebral disc degeneration. **(A)** Histological images of (Control and degenerated) intervertebral discs stained with H&E, Masson, and SOFG. **(B)** Immunofluoresence images (representative) which show the expression pattern of CD36, SESN3, and HMOX1 of disc tissues of (control and IDD) rats. **(C)** Quantification of immunofluorescence intensity. **(D)** Histological grading of Control and IDD group. **(E–G)** The expression of CD36, SESN3, and HMOX1 in normal and degenerated intervertebral discs of rats. *p < 0.05, **p < 0.01, ***p < 0.001.

## 4 Discussion

Low back pain and the associated functional impairment are mainly caused by the degenerative changes that are taking place in the intervertebral discs, which is commonly known as intervertebral disc degeneration (Mohd Isa et al., 2022; Zehra et al., 2022).

However, the mechanisms underlying this pathology are not completely elucidated. Recently oxidative stress has turned out to be one of the key contributors of disc degeneration, which leads to matrix degradation, induces cell aging and stimulates the inflammatory cascades (Gao et al., 2024). But the exact oxidative stress related genes involved in IDD and how these genes work together in the local immune microenvironment have not been fully clarified (Lan et al., 2022; Zheng et al., 2025). Thus, we combined different transcriptomic datasets and applied a whole analytic method involving the differential expression analysis, the consensus clustering, WGCNA, immune infiltration evaluation and the experimental verification to find out the genes related to oxidative stress in IDD. We have shown that CD36, SESN3 and HMOX1 might be the key genes who act as central regulators between oxidative stress and immune modulations in the degenerative disc. This work gives us several important observations.

An extensive check-up of open-access transcriptomic data has shown that degenerated nucleus pulposus tissues have a special transcriptional signature related to oxidative stress. The use of consensus clustering techniques has enabled the finding of two different molecular subtypes, which supports the idea of biological heterogeneity related to oxidative stress in IDD. Finally, the use of Weighted Gene Co-expression Network Analysis (WGCNA) together with differential expression analysis based on clusters, has allowed to find three solid hub genes: CD36, SESN3 and HMOX1. These genes are related to lipid metabolism, cellular stress reactions and antioxidant mechanisms, therefore it seems that the oxidative imbalance that can be found in IDD could be the expression of a synchronous derangement of both metabolic and cytoprotective pathways.

The biological relevance of these specific genes is high. Indeed, CD36 works as a universal scavenger receptor involved in fatty acid absorption, in the processing of lipids, in inflammatory responses and oxidative injury (Guo et al., 2024; Kim et al., 2024; Li M. et al., 2026). In previous works in many different types of tissues, it has been shown that CD36 is able to potentiate the production of ROS and to promote inflammatory signaling pathways and thus to aggravate the chronic tissue damage (Hou et al., 2021; Yang and Silverstein, 2019). We found a positive association of CD36 levels with resting mast cells, which could indicate a possible link between the strain of the metabolism and the activation of the immune system in the degenerating intervertebral discs. Finally, SESN3, which is a member of the Sestrin family, is well known for his contribution in antioxidant protection and to the metabolic regulation in stress condition (Hagenbuchner et al., 2012; Zamkova et al., 2013). The negative correlation we have found between SESN3 and the level of naive CD4+T cells in this study could indicate that the redox sensitive mechanisms could be involved with the local immune modulation in the context of the disc degeneration. HMOX1 which encodes Heme oxygenase 1 is a classic stress-inducible enzyme which is endowed with abilities to protect the cells, fight oxidative damage and modulate the immune responses (Cai et al., 2025; Liang et al., 2025; Peng et al., 2025). According to our data, we found an important correlation between the level of expression of HMOX1 with several different population of immune cell as for rest mast cells, M0 macrophage, activated natural killer cell, as well as naive CD4^+^ T and B lymphocyte. This may indicate that HMOX1 could be important in the link between the oxidative stress response with the immune adaptation in the micro environment of the degenerating intervertebral disc.

An important part of this work is to study the patterns of immune cell infiltration related to the intervertebral disc degeneration (IDD). While the intervertebral disc was thought to be an immune-privileged site, the data accumulated so far suggest that the immune involvement becomes progressively more important the more degenerative processes progress. We found that there are different variations of resting mast cells and of the T cell subpopulations between the different molecular subtypes related to the oxidative stress and between different stages of the disease. Moreover, each of the three main genes was strongly correlated to some particular groups of immune cells. These results are in favour of the hypothesis that the oxidative stress in IDD is not an isolated event, but it is also intimately connected to the re-structuration of the immune microenvironment. Such interactions could be at the basis of the sustained chronic inflammation, of the accelerated ECM breakdown and of the gradual tissue deterioration. Beside the identification of potential biomarkers, we also built the predicted ceRNA networks and the transcription factor-mRNA regulatory frameworks to. If we look at the upstream regulatory architecture of the above key genes, we can see that most probably a complex interplay among different miRNAs, lncRNAs and transcription factors regulate the expression of the CD36, SESN3 and HMOX1. Although these proposed networks are still predictive in the present state and not yet confirmed in a mechanistic way, they provide a very important base for the next studies to understand the upstream regulatory mechanisms involved in the IDD.

The present research has a number of advantages. The use of a variety of independent datasets was used to cancel the biases in the analyses done in single cohorts and thus improve the robustness of the candidate gene identification. Additionally, the credibility of our results was further supported by the combination of the computational screening with the experimental validation in both human tissue samples and in an *in vivo* rat model. However, there are also some limitations to be considered. Firstly, the aggregeted sample size from the public databases was not that large, so larger external cohorts were required to check the generalizability of our results. Secondly, the landscape of the immune infiltration was estimated by the computational methods of deconvolution and not measured directly. The future investigations with single-cell RNA sequencing or spatial transcriptomics would provide more convincing evidences about the specific identity and the spatial localization of immune cell populations in the degenerative discs. Thirdly, we only confirmed the expression patterns of the hub genes, but not their direct function.

However, targeted mechanistic investigations are still needed to really clarify those specific functions involved in the modulation of disc cell senescence, extracellular matrix metabolism, and crosstalk with the immune system. Some constraints concerning this investigation should be pointed out. First, the cohort is rather small and there is no independent check from other sources. Since clinical corroborative data are very scarce, the purpose of this work is to supply information coming from bioinformatics analyses. Moreover, the two different molecular subtypes defined by consensus clustering have not been yet deeply studied or defined in the general biological and clinical research field. Finally, the estimation of the abundances of immune cells have been done only with the CIBERSORT computational tool and no confirmation has been done by histological examination or flow cytomertic analysis. This is a methodological shortcoming that could prevent an accurate elucidation of alterations within the immune microenvironment; so, next investigations should include multimodal for the validation.

In the future, we plan to perform some comparative studies using well known clinical parameters, such as the age of the patient or the Pfirrmann grading, and using as well the activity of the path way as, for example, GSVA. The goal is to show that the subtypes found are really corresponding to specific profiles of oxidative stress or immune status. It should be also noticed that the proportions of immune cells were estimated with the CIBERSORT algorithm without confirmation by a histologic examination or flow cytometry. Another important limitation of this work is that a lot of results, such as the regulatory networks with the predicted ceRNAs, are from the bioinformatic modelling and need to be verified by other empirical results. Independently of these limits, we think that the present work provides interesting points of view for future scientific work and these limits should be corrected in the following works.

In conclusion, this work has systematically identified CD36, SESN3 and HMOX1 as core genes related to oxidative stress in the context of IDD. It was also shown that the three genes are well correlated with the changes that happens in the immune microenvironment. These results add to the knowledge about the molecular crosstalk between inflammatory processes and oxidative stress during the disc degeneration. They could help to design the future therapeutic approaches and diagnostic biomarkers.

## Data Availability

The original contributions presented in the study are included in the article/Supplementary Material, further inquiries can be directed to the corresponding authors.
